# Multi-Sacrificial Bonds Enhanced Double Network Hydrogel with High Toughness, Resilience, Damping, and Notch-Insensitivity

**DOI:** 10.3390/polym12102263

**Published:** 2020-10-01

**Authors:** Manxi Sun, Jianhui Qiu, Chunyin Lu, Shuping Jin, Guohong Zhang, Eiichi Sakai

**Affiliations:** 1 Department of Mechanical Engineering, Faculty of Systems Science and Technology, Akita Prefectural University, Akita 015-0055, Japan; D20S003@akita-pu.ac.jp (M.S.); D21S005@akita-pu.ac.jp (C.L.); zhang@akita-pu.ac.jp (G.Z.); e_sakai@akita-pu.ac.jp (E.S.); 2 College of Chemistry and Chemical Engineering, Hexi University, Zhangye 734000, China; chem2011@hxu.edu.cn

**Keywords:** double network ionic hydrogel, multi-sacrifice bonds, high toughness, fast recovery, notch-insensitivity

## Abstract

The engineering applications of hydrogels are generally limited by the common problem of their softness and brittlness. In this study, a composite double network ionic hydrogel (CDN-gel) was obtained by the facile visible light triggered polymerization of acrylic acid (AA), polyvinyl alcohol (PVA), and hydrolyzed triethoxyvinylsilane (TEVS) and subsequent salt impregnation. The resulting CDN-gels exhibited high toughness, recovery ability, and notch-insensitivity. The tensile strength, fracture elongation, Young’s modulus, and toughness of the CDN-gels reached up to ~21 MPa, ~700%, ~3.5 MPa, and ~48 M/m^3^, respectively. The residual strain at a strain of 200% was only ~25% after stretch-release of 1000 cycles. These properties will enable greater application of these hydrogel materials, especially for the fatigue resistance of tough hydrogels, as well as broaden their applications in damping.

## 1. Introduction

Hydrogels are wet and soft materials that have drawn great attention in recent years thanks to their promising applications, and have been widely used as scaffolds for tissue engineering [1], vehicles for drug delivery [2], wearable electronics, and in hydrogel-based soft machines [3]. Mechanically strong and tough hydrogels are the first choice for artificial tissues, but most hydrogels are not adequate because of the demanding requirements of such applications [4]. For example, hydrogel-made knee cartilage is expected to sustain peak stresses of 4 to 9 MPa for 1 million cycles per year [5]. Moreover, the hydrogel-made skin needs to meet the a high stiffness level of ~100 MPa, high fracture energy of ~3600 J/m^2^, and high water content of 40–70 wt.% [6,7]. When hydrogels are used in robotic arms, repeated stretching and immediate restoration must be possible [8]. Moreover, engineering hydrogels rarely possess both high stiffness and toughness, because hydrogels usually become brittle when excess crosslinkers are added to make them stiff [9]. These brittle hydrogels are notch-sensitive, that is, their high stretch ability and strength decrease markedly when the samples contain notches or any other features that cause inhomogeneous deformation [10]. This factor greatly limits their applications. Specifically, general hydrogel performs poorly under prolonged static and cyclic loads. Overall, the existing hydrogels tested so far have difficultly providing these comprehensive mechanical properties at the same time.

Several strategies with different mechanisms of energy dissipation have been developed to synthesize tough hydrogels, including double network (DN) hydrogels, nano- and micro-composite hydrogels, and tri-block copolymers and hydrophobic associated hydrogels [11,12,13,14,15]. The fracture energy of hydrogels has been enhanced by orders of magnitude from 10 to 10,000 J/m^2^ [10]. Among them, the high toughness of DN hydrogel attributes the interpenetration between the first brittle and second soft networks. When a DN hydrogel is stretched, the sacrificed first brittle network ruptures and dissipates energy, while the second soft network retains elasticity. However, when the sample is subjected to excessive forces, conventional DN hydrogel suffers from internal network fractures and irreversible deformation due to the fracture of chemical sacrificial bonds, which leads to poor recovery and anti-fatigue properties [16,17]. Therefore, sacrificing reversible noncovalent bonds instead of covalent bonds has been advocated. Zheng et al. [18] reported a novel tetra-polyethylene glycoltetra PEG/reduced graphene oxide nanocomposite hydrogel with a fracture energy of ~200 J/m^2^, tensile strength of ~700 kPa, and Young’s modulus of ~120 kPa. Gao et al. [19] prepared a high strength hydrogel with core-shell hybrid nanoparticles made of cross-linked polyacrylamide as the first network and Ca^2+^ cross-linked alginate as the second network, featuring a fracture stress of ~1 MPa, Young’s modulus of 54 kPa, and toughness of ~10 MJ/m^3^. However, these improvements are still limited.

In this work, we synthesized a composite polyvinyl alcohol (PVA)/poly (acrylic acid) (PAA)/silicone hydrogel (tensile strength of ~21 MPa, fracture elongation of ~700%, Young’s modulus of ~3.5 MPa, and toughness of ~48 MJ/m^3^) via visible-light-trigger polymerization and the introduction of dynamic physical ionic bonds. Moreover, this hydrogel exhibited high resilience, damping, and notch-insensitivity. In this study, hydrolyzed silane (silanol) was grafted onto PAA/PVA chains to create a dynamic chemical crosslinking network. In this way, numerous hydrogen bonds were formed among the hydrophilic groups, and the hydrogen bonds enhanced by ions formed reversible physical crosslinking. In this way, a tough hydrogel was obtained. When the hydrogel was stretched, the reversible fracture-reorganization of the hydrogen bonds dissipated the energy, which significantly improved the resilience of the hydrogel. This work provides new clues for designing hydrogels with excellent comprehensive mechanical properties.

## 2. Materials and Methods

### 2.1. Preparation of Hydrogel

Firstly, 10 wt.% of triethoxyvinylsilane (TEVS, Sigma-Aldrich, Tokyo, Japan) was added to deionized water at room temperature and vigorously stirred for 12 h until a transparent dispersion solution of vinyl silanetriol (VSTO) solution was obtained.

Acrylic acid (AA, Nacalai Tesque, Kyoto, Japan), 0.5 wt.% photo-initiator camphorquinone (CQ, Tokyo Chemical Industry, Tokyo, Japan) based on AA, and VSTO (0, 0.1, 0.5, 1, 5 wt.% based on AA) were sequentially added into a 10 wt.% polyviny alcohol (PVA, degree of polymerization of 2000, Nacalai Tesque, Kyoto, Japan) solution. The mass ratio of PVA/AA was kept at 1:9. The mixture solution was stirred for 1 h to achieve homogenization and then degassed under a vacuum for 10 min. Afterward, the obtained precursor solution was slowly poured into a transparent mold (100 mm × 100 mm × 2 mm). Then, the precursor solution was visible-light-trigger polymerized for 30 min using a visible light source (LS-M210, Sumita, Saitama, Japan). Finally, the polymerized double network hydrogels were immersed in a saturated LiCl (Nacalai Tesque, Kyoto, Japan) solution for 8 h to prepare the composite double network ion hydrogel (CDN-gel). The CDN-gels were named GEL-X, where X denotes the weight ratio of the TEVS to AA, for example, GEL-0, GEL-0.1, GEL-0.5, GEL-1, and GEL-5.

### 2.2. Characterization

The morphologies of the CDN-gels were characterized by a scanning electron microscope (SEM, S-4300, Hitachi, Tokyo, Japan). Before characterization, the hydrogel samples were prepared via the freeze-drying method, and then all samples were sputter-coated with platinum to provide enhanced conductivity.

The mechanical properties of the CDN-gels were tested using a universal testing machine (Instron 3300, Instron, Norwood, MA, USA). For the tensile mode, the CDN-gel was cut into a size according to the standard of JIS K6251, and the CDN-gel with a diameter of 8 mm and a height of 2 mm was used in compression mode. A dynamic mechanical analysis (DMA, RSA-G2, TA Instrument, New castle, DE, USA) was used to measure the storage modulus and loss modulus of the CDN-gels at a frequency from 1 to 100 Hz under constant strain amplitude (1%). Before measurement, the CDN-gels (8 mm in diameter and 2 mm in thickness) were subjected to an axial force (0.981 N). All of the mechanical characterizations of hydrogels were conducted in an indoor environment (25 °C, relative humidity (RH) = 55%).

The damping ability of the CDN-gel was evaluated by a homemade evaluation device, whose details have been described in our previous work [20]. The CDN-gel was placed under a vibration source (Present mixer 2013, Taitec, Saitama, Japan) as a shock-absorbing material, and the signal change caused by the shock of the vibration source was recorded by a force sensor placed under the hydrogel to evaluate the damping ability of the CDN-gel.

The notch-sensitivity of the CDN-gels was analyzed by calculating the change in the fracture energy using a method introduced by Rivlin and Thomas [5,21]. The CDN-gels (width *a_0_* = 50 mm and thickness *b_0_* = 2 mm) with 4 mm long notches (processed by a razor blade) were used to evaluate the notch-sensitivity when the gels were stretched, and the non-notched sample was pulled to measure the force–length curve. During stretching, the distance between the two clamps was *L_0_* = 10 mm, and the cross-head speed was 30 mm/min. When the two clamps were pulled to distance *L*, the area beneath the force–length curve revealed the results of the applied force *W(L*). The notched sample was then pulled, and the critical distance (*L_c_*) was recorded when the notch turned into a running crack (Figure S1). The fracture energy was calculated using Equation (1):

*Γ* = *W*(*L_C_*)/(*a*_0_·*b*_0_) (1)

## 3. Result and Discussion

### 3.1. Synthesis of the CDN-gel

**Figure 1:**
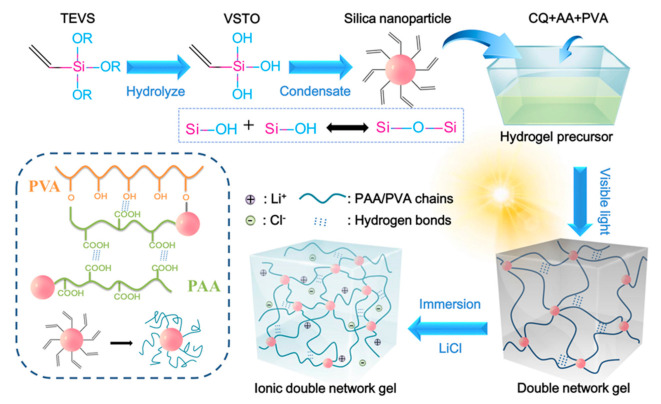
Schematic diagram of the preparation process and network structure of the composite double network ionic hydrogel (CDN-gel). PVA, polyviny alcohol; PAA, poly (acrylic acid); TEVS, triethoxyvinylsilane; VSTO, vinyl silanetriol; AA, acrylic acid; CQ, camphorquinone.

Visible light is a safe, low cost, and easily acquired trigger that has attracted attention in synthesis of polymers via radical-initiated polymerization in recent years [22,23]. Figure 1 presents the preparation process and interactions of the CDN-gel. The alkoxy groups are first hydrolyzed into silanol groups in the presence of water, and the silanol groups then condensate to form a siloxane bond [24,25]. The free-radical grafting polymerization mechanism of the PVA/PAA hydrogel triggered by visible light with a CQ initiator was proposed in our previous work [20]. Specifically, CQ uses light to dissociate the initiator molecules into free radicals, which react with double bonds in the monomers or pre-polymers, thus allowing crosslinking to occur. In this work, monomer AA not only grafted with VSTO, leading to the formation of nanobrush gelators, but was also polymerized on the PVA backbone, resulting in a polymer network with a grafted structure. Simultaneously, the silanol can be adsorbed to the PVA on its OH-rich chains through hydrogen bonding, further leading to siloxane bridges [25]. Finally, the visible light-induced hydrogels were exposed to a saturation lithium solution to increase the crosslinking sites between the polymer chains by metal doping. The CDN-gels created by this method were expected to have a network structure constructed through a combination of ion-mediated reversible physical cross-linking, intra- and inter-polymer chain hydrogen bonding, physical entanglement of the polymer chains, and covalent cross-linking.

### 3.2. Morphology of the CDN-Gel

**Figure 2:**
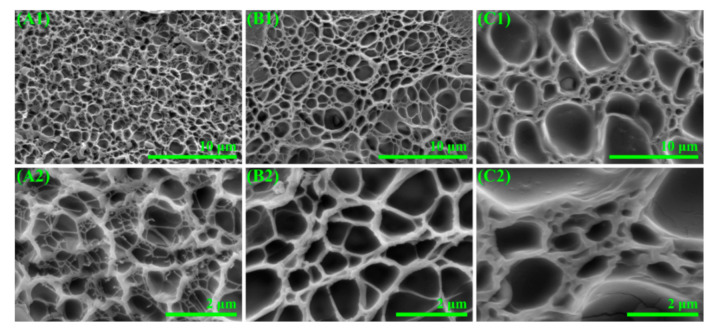
Broken sections of CDN-gels. (**A1**,**A2**) GEL-0; (**B1**,**B2**) GEL-0.5; (**C1**,**C2**) GEL-5.

Figure 2 shows SEM images of the inner structure of the freeze-dried CDN-gels with different VSTO ratios. The inner structures of the CDN-gels presented a sponge-like morphology, similar to most lyophilized gels [26,27]. As with the introduction of VSTO, the structures of the CDN-gels changed significantly as a result of strong interactions. For GEL-0 (Figure 2A), a porous main network structure and a relatively finer mesh structure were observed. When the VSTO was introduced into the CDN-gel, the finer mesh structure disappeared and an interconnected, uniform, and complete network structure was formed (GEL-0.5, Figure 2B). However, an increased pore size difference was clearly observed on GEL-5 (Figure 2C).

The introduction of VSTO can have two effects on the crosslinking network of the CDN-gels. On the one hand, VSTO as a multifunction cross-linking agent can increase the crosslinking density of the CDN-gels and contribute to the formation of a uniform porous network structure [28]. On the other hand, excessive VSTO is distributed in a disordered manner throughout the whole network and causes the chain links to become different in length. Moreover, the regularity of molecular chains is also destroyed by an increase in cross-linking points [29].

It is believed that the swelling capacity of hydrogel reflects the homogeneity of its network [30]. The water contents of all CDN-gels showed a tendency to increase first and then stabilize over time (Figure S2). Notably, the equilibrium of the moisture content of the CDN-gels showed a trend of increasing first and then decreasing with an increase of VSTO. Indeed, an appropriate amount of VSTO can facilitate the formation of a more complete and uniform network structure and increase the flexibility of the molecular chains [31,32]. A large amount of VSTO would lead to more new polymerization chains, making the length of the segment between the cross-linking points shorter and more dense. The elastic contraction force that hinders the swelling of the CDN-gel thus increases sharply, the network space of the CDN-gel becomes smaller (Figure 2C), and the free water decreases (Figure S2). In general, the elastic interconnected network structure being enhanced by the correct amount of VSTO indicates that more effective energy dissipation could be achieved, resulting in an improvement of the mechanical properties and elasticity of the hydrogels.

### 3.3. High Strength and Toughness of CDN-Gels

**Figure 3:**
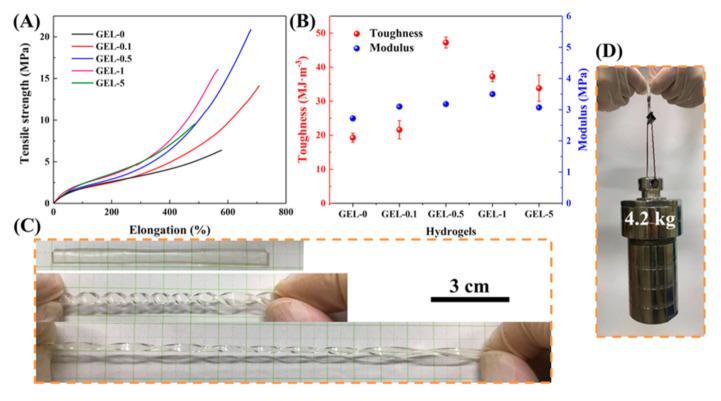
(**A**) Tensile curves of CDN-gels; (**B**) toughness and Young’s modulus of CDN-gels; (**C**) twisting and stretching after being twisted for GEL-0.5; and (**D**) carrying a heavy steel block weighing 4.2 kg using GEL-0.5 (width = 4 mm, thickness = 2 mm).

The mechanical properties of CDN-gels were investigated using tensile tests (Figure 3). It can be seen that GEL-0 presented relatively poorer mechanical performance (a tensile strength of ~6 MPa, elongation at break of ~600%, toughness of ~20 MJ/m^3^, and Young’s modulus of ~2.8 MPa) than the other CDN-gels, despite offering better performance than most reported hydrogels in the literature (Figure S3). Significant enhancements in mechanical performance were observed for GEL-0.1, -0.5, -1, and -5. Notably, the tensile strength, elongation at break, toughness, and Young’s modulus of the optimal CDN-gel (GEL-0.5) reached ~21 MPa, ~700%, ~48 MJ/m^3^, and 3.5 MPa, respectively. Moreover, the as-synthesized GEL-0.5 was further observed to withstand different deformations, such as twisting and large stretching after twisting (Figure 3C). Figure 3D illustrates that GEL-0.5 was strong enough to lift a hydrothermal reactor autoclave weighing 4.2 kg. In general, the synergy between the multiple cross-linking points gives GEL-0.5 excellent mechanical performance.

The mechanical performance of GEL-0 was explained in our previous work [20] (i.e., a double cross-linked hydrogel with dynamic physical cross-linking (ionic bonding and hydrogen bonding) and chemically cross-linked PVA/PAA networks). Herein, the introduction of VSTO (multiple function covalent cross-linking agents) into GEL-0 resulted in further significant improvements in mechanical strength, extensibility, toughness, and resilience (Figure 3A or Figure S4). During the stretching process, the hydrogen bonds in the CDN-gels network dissipate energy through reversible break-reformation and homogenize the network. VSTO can maintain the elasticity of the network and act as a transfer center to homogenize the stress distribution in the network [33]. The applied stress is then absorbed and redistributed by the VSTO, thus the crack propagation is delayed by the numerous grafted PAA/PVA chains. However, too much VSTO (GEL-5) will increase the number of initiation points for polymer growth for a fixed AA monomer concentration. Consequently, the average polymer length will be reduced, thereby reducing the flexibility of the PAA/PVA chains.

Similar phenomena have been reported by M. Zhong, et al. [34]. In that work, vinyl-hybrid silica nanoparticles (VSNP) were used as covalent cross-linking agents with ferric ions as ionic crosslinkers, ultimately obtaining tough and stretchable nanocomposite ionic cross-linked VSNP/PAA physical hydrogels. Nevertheless, the mechanical performance of our GEL-0.5 is still much higher than that of the VSNP/PAA hydrogel (tensile strength 860 kPa, elongation at break ~2300%).

### 3.4. Excellent Recoverability of GEL-0.5

#### 3.4.1. Cyclic Tensile Tests

**Figure 4:**
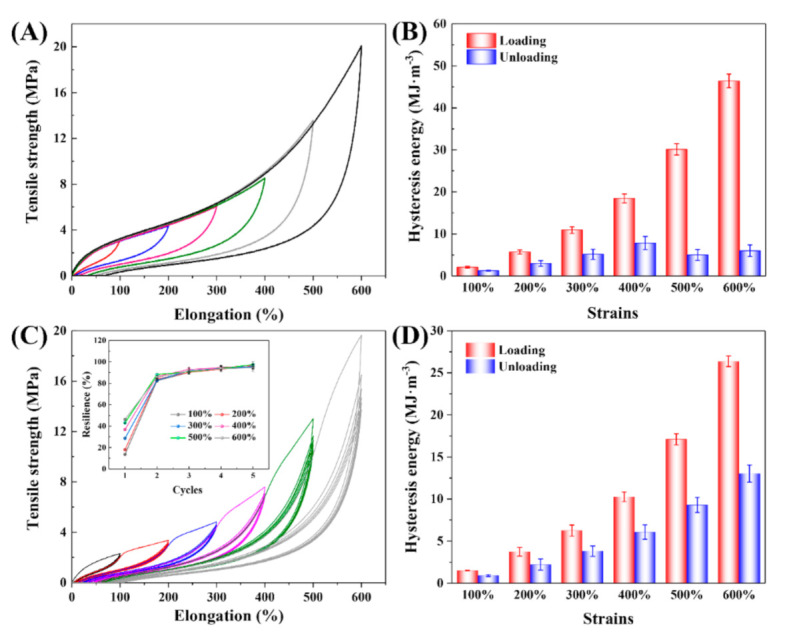
(**A**) Loading–unloading curves of GEL-0.5 stretched for one cycle under different strains (new GEL-0.5 for every cycle); (**B**) the calculated hysteresis energy during the cyclic tensile tests in (**A**); (**C**) loading–unloading responses of the same GEL-0.5 submitted to gradient increasing strain (increase the strain after 6 cycles under every fixed strain); and (**D**) the calculated hysteresis energy of (**C**) during the first cycle of the softening process.

Tensile cyclic loading–unloading tests were conducted to evaluate the hysteresis behaviors of GEL-0.5 (Figure 4) during the stretching fracture process of the gel samples [35,36]. For the successive cyclic loading–unloading tests of the new GEL-0.5 at various strains ranging from 100 to 600% (Figure 4A), the dissipated energy of GEL-0.5 increased with the deformation strain (Figure 4B). For the gradient cyclic tensile test (Figure 4C), the subsequent cyclic loading–unloading curves (2–6 cycles) remained almost overlapping and presented similar dissipated energies (resilience > 80%, inset figure in Figure 4C) after the first stretching–releasing cycle. The observed material softening phenomenon, whereby a lower resulting stress appears after the first load and the hydrogel response curves coincide during the following cycles at the same applied strain, could be explained by the Mullins effect [16,37]. The softening increases progressively with the maximum strain (Figure 4C), and the dissipated energies of the first cycle of softened GEL-0.5 at different strains were smaller than those of the unsoftened GEL-0.5 (Figure 4B,D). Moreover, it can be observed from the first loading curves under each strain of the repeatedly stretched GEL-0.5 (Figure 4C) that they nearly continued to increase along the trajectories of the previous tensile test, thus indicating the excellent resilience of GEL-0.5.

#### 3.4.2. Cyclic Compressive Tests

**Figure 5:**
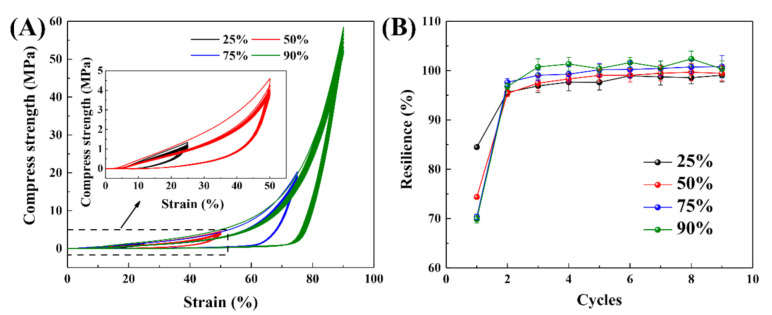
(**A**) Loading–unloading curves of GEL-0.5 during successive compression cycles at different strains; (**B**) the calculated resilience of GEL-0.5 during the cyclic tensile tests.

The compression cycle results of GEL-0.5 are presented in Figure 5, which shows basic stable stress–strain curves. GEL-0.5 presented a compressive strength of ~60 MPa at a strain of 90% and the ability to rapidly return to its original position. As the strain increased, the network structure of GEL-0.5 changed slightly. The curves of the second cycle were observably inconsistent with those of the first cycle when the compression strain was over 50%, indicating that an overly large deformation could cause irreversible damage to the network structure. Nevertheless, the resilience of GEL-0.5 was still above 70% (from the first cycle) at a compressive strain of 90% and reached a stable and high value from the second cycle (>95%), as shown in Figure 5B. Moreover, the stable hysteresis energy dissipation of GEL-0.5 could be attributed to both the dissociation of ionic and hydrogen bonds and the friction between polymer chains [38,39]. After the applied stress was removed, the sacrificial bonds were able to reform rapidly, and thereby restore the integrity of GEL-0.5. Above all, GEL-0.5 exhibited excellent compressive properties during successive process cycles.

### 3.5. High Recovery Properties

**Figure 6:**
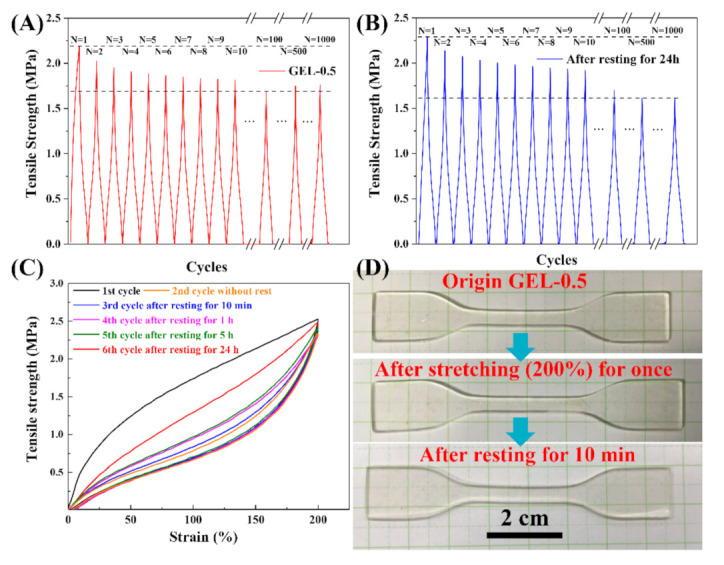
(**A**) Cyclic tensile curves of GEL-0.5 obtained after 1000 tests at a strain of 200%; (**B**) the same operation as (**A**) was performed after GEL-0.5 rested for 24 h; (**C**) cyclic stress–strain curves of GEL-0.5 at a 200% strain with different rest times; and (**D**) digital photos of GEL-0.5 with fast resilience.

Most of the reported hydrogels possess obviously time-dependent recovery properties that significantly increase with an increase in rest time [40]. To further investigate the recovery properties of GEL-0.5, several tensile operations were conducted, as shown in Figure 6. After being stretched 1000 times at a strain of 200%, the maximum stress of each cycle decreased from 2.2 to 1.7 MPa (Figure 6A). After GEL-0.5 rested for 24 h at room temperature, the reloading curves were almost the same as those for the first 1000 cycles (Figure 6B). The observed slight increase in maximum stress could be due to self-healing of the broken ionic and hydrogen bonds [41]. Figure 6C illustrates the recovery speed of GEL-0.5. It can be observed that GEL-0.5 was much weaker when the second load was applied immediately and recovered somewhat if the second load was applied one day later. GEL-0.5 showed negligible hysteresis, and the sample fully recovered its original length after resting for 10 min (Figure 6D). The pronounced hysteresis and relatively small permanent deformation (<25%) of GEL-0.5 were further demonstrated by loading–unloading tests (20 times) at a strain of 200% (Figure S4). In a word, the GEL-0.5 has an excellent fatigue resistance and rapidly recovery ability by introducing multiple sacrificial bonds.

### 3.6. Dynamic Mechanical Analysis

The liquid phase of hydrogel is constrained within its three-dimensional network, thus producing visco-elastic properties [42,43]. To investigate the influence of VSTO on the viscoelasticity of CDN-gels, a DMA was conducted from 1 to 100 Hz. Meanwhile, the storage modulus *E*′ (a measure of elasticity and stiffness), the loss modulus *E*″ (a measure of viscous), and *Tan δ* (*E*″/*E*′) were measured.

**Figure 7:**
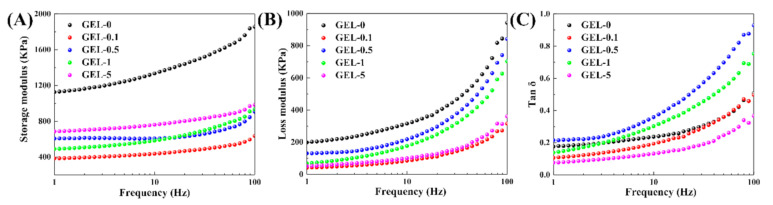
Viscoelasticity of CDN-gels. (**A**) Storage modulus; (**B**) loss modulus; (**C**) tan δ.

As shown in Figure 7, *E*′ is always higher than *E*″ in the frequency range of 1–100 Hz, which indicates that the CDN-gels have considerable strength to withstand pressure and resist certain impacts. Moreover, both the *E*′ and *E*″ of all CDN-gels increased as the frequency increased, indicating that the energy dissipation caused by intermolecular friction was proportional to the frequency. For the *E*′ of CDN-gels at 1 Hz (*E*′_1_), the *E*′_1_ dropped drastically after the VSTO was added and then gradually increased from 400 to 700 KPa with the VSTO content. It was reported that the *E*′ in the low frequency relates to the effective network chain density (*N*), which can be depicted as follows [38]:

*E*′ = *λNRT*, (2)

where *λ* is the hydrogel-based constant and *R* and *T* are the gas constant and absolute temperature, respectively. For the CDN-gels with VSTO, the increased *E*′ can be explained by the increase in the effective network chain density with VSTO content, while the drastic decrease of *E*′ after the VSTO was introduced could be attributed to a change in *λ*, which reflects a large change in the flexibility of molecular chains. An excessive amount of VSTO (GEL-5) would limit the movement of polymer chains, resulting in a decrease in viscosity (reflects by *E*″, Figure 7B). *Tan δ*, also known as damping, is determined by the ratio of the storage modulus and loss modulus [44]. After introducing VSTO into CDN-gels, GEL-0.5 presented the highest *tan δ* from 1 to 100 Hz (Figure 7C). The high *tan δ* of GEL-0.5 reflects moderate viscoelasticity, which indicates its potential application in the field of vibration absorption.

### 3.7. Damping Ability

**Figure 8:**
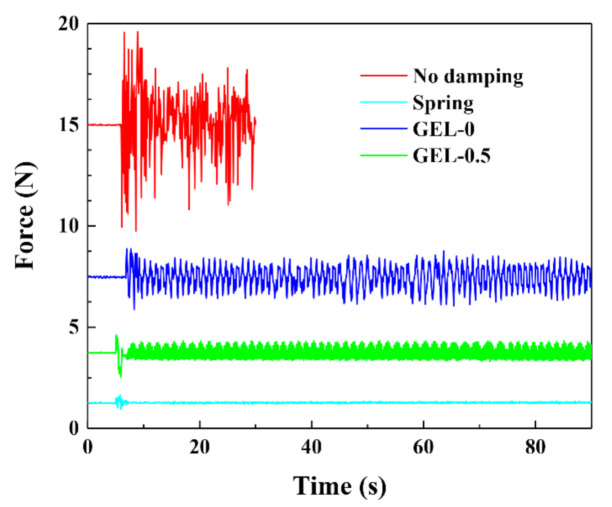
Comparison of the damping effect of different vibration dampers.

Figure 8 shows the vibration signals after using different shock absorption materials as a buffer, while Movie S1 presents the vibration processes. When no damping material was placed, the vibrator beat violently on the metal plate, and the amplitude of the detected force was ~5 N. Under shock absorption, the force signals quickly converged to a stable range after experiencing a large shock at the moment when the power was turned on. The force amplitude of the spring, a traditional excellent shock absorption material, reduced to ~0.05 N. In this work, the CDN-gels exhibited certain damping abilities. For GEL-0, the force amplitude was still high (~1 N) and not very stable. After the addition of VSTO, GEL-0.5 reflected much better damping ability (~0.4 N) than GEL-0.

Indeed, the damping ability of the CDN-gel mainly depends on its elasticity. A stiff hydrogel cannot absorb enough of the shock, while a soft hydrogel does not have a sufficient response speed to resolve vibrations within a certain frequency [45,46]. The excellent elasticity and recovery properties of GEL-0.5 provide it with good damping ability. As a shock absorbing material, hydrogel has the advantages of higher quality and space reduction over metal springs, although the damping ability of hydrogel is still not as good as that of metal springs.

### 3.8. Notch-Insensitive Properties

Elastic hydrogel is known to be brittle and notch-sensitive. That is, the stretchability and strength of this material decrease markedly when the samples contain notches or any other features that cause inhomogeneous deformation [47]. In this work, different dynamic physical crosslinks (hydrogen and ionic bonds) of hydrogel were shown to effectively dissipate energy through the fracture-reformation process during stretching. Moreover, VSTO, a multifunctional crosslinking point, can make the network uniformly stressed to reduce the stress concentration [34]. Therefore, CDN-gels are expected to have good notch-insensitive properties.

**Figure 9:**
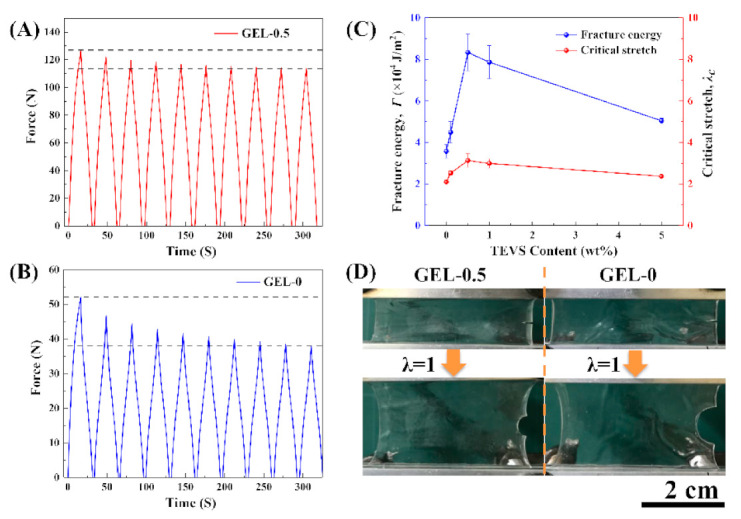
(**A**) Cyclic tensile curves for 10 cycles of GEL-0.5 with single-notch (4 mm) at a strain of 100%; (**B**) cyclic tensile curves for 10 cycles of GEL-0 with a single-notch (4 mm) at a strain of 100%; (**C**) the ratios of the fracture critical stretch (λ) and the fracture energy (Γ) of the CDN-gels; (**D**) photos of notch deformation of GEL-0 and GEL-0.5 during stretching.

When stretching the CDN-gels with notches (Figure 9), the notches on the edges of the CDN-gels are the domain where stress concentration can easily occur. GEL-0.5 was shown to have better tensile cycle stability and higher uniaxial stress (120 N) stability than GEL-0 (40 N) (Figure 9A,B), indicating better resistance to cracks or notch expansion in GEL-0.5. Under a strain of 100%, the notch in GEL-0.5 did not expand significantly during the stretching process (Figure 9D). Figure 9C presents the changes in the highest fracture energy and critical stretch with the VSTO contents. Compared with the non-notched CDN-gels, the notched CDN-gels can withstand less deformation, but can still be stretched to 2–3 times their original length, presenting excellent stretchability and notch-insensitive properties. The highest fracture energy can be obtained by integrating the stress–strain curves of non-notched CDN-gels at the corresponding elongation. The notched GEL-0.5 presented the highest fracture energy (Figure 9C), which is several orders of magnitude similar to natural rubber (~104 J/m^2^) [48]. For the notched GEL-5, the PAA/PVA chains were densely crosslinked with a high concentration of VSTO. Only a small zone around the root of the notch was stressed enough to break the PAA/PVA chains (hard and brittle), so the fracture energy was low.

Overall, VSTO can strengthen the network of CDN-gels, increase the gels’ density, and contribute to forming cross-linking points, allowing the gels to bear more stress and dissipate more energy. As a result, VSTO can prevent crack propagation more effectively.

## 4. Conclusions

In this work, we presented a simple method to synthesize a composite double network ionic hydrogel by visible light triggering polymerization and salt impregnation. The VSTO, as a multi-crosslinking point, not only introduces an energy dissipation unit, but also homogenizes the network of CDN-gels. The right amount of VSTO can significantly improve the mechanical properties and swelling ratio of CDN-gel. The tensile strength, fracture elongation, modulus, and toughness of the optimized CDN-gel (GEL-0.5) can reach up to ~21 MPa, ~700%, ~3.5 MPa, and ~48 MJ/m^3^, respectively. GEL-0.5 exhibited stable mechanical properties in repeated tensile and compression tests thanks to its excellent resilience. The damping test results show that the resilient GEL-0.5 has a good shock absorption property. Moreover, GEL-0.5 exhibits a certain ability to resist crack propagation. Overall, this kind of hydrogel with excellent comprehensive mechanical properties is expected to be used as artificial tissues, such as cartilage and achilles tendon.

## Supplementary Materials

The following are available online at https://www.mdpi.com/2073-4360/12/10/2263/s1, Figure S1: Schematic diagram of calculating *W(L_c_)*; Figure S2: Water absorption ability of CDN-gels; Figure S3: Comparison of CND-gels and various soft materials obtained from literatures in tensile strength and elongation; Figure S4: Cyclic tensile tests of (A) GEL-0 and (B) GEL-0.5 at strain of 200%; Movie S1: Vibration processes with different damping materials.
